# Efficacy of Removing Bacteria and Organic Dirt from Hands—A Study Based on Bioluminescence Measurements for Evaluation of Hand Hygiene When Cooking

**DOI:** 10.3390/ijerph18168828

**Published:** 2021-08-21

**Authors:** Octavian Augustin Mihalache, Daniela Borda, Corina Neagu, Paula Teixeira, Solveig Langsrud, Anca Ioana Nicolau

**Affiliations:** 1 Faculty of Food Science and Engineering, Dunarea de Jos University of Galati, Domnească Street 111, 800201 Galati, Romania; octavian.mihalache@ugal.ro (O.A.M.); daniela.borda@ugal.ro (D.B.); corina.neagu@ugal.ro (C.N.); 2 CBQF-Centro de Biotecnologia e Química Fina-Laboratório Associado, Escola Superior de Biotecnologia, Universidade Católica Portuguesa, Rua Diogo Botelho 1327, 4169-005 Porto, Portugal; pcteixeira@porto.ucp.pt; 3 Norwegian Institute of Food, Fisheries and Aquaculture Research (Nofima), Osloveien 1, N-1430 Ås, Norway; solveig.langsrud@nofima.no

**Keywords:** cleaning, washing, rinsing, greasy hand, soap, wipe

## Abstract

The objective of this study was to evaluate the efficacy of dirt removal (bacteria and organic matter) of several hand-cleaning procedures. The results from the hand hygiene experiment indicated that washing hands with warm water and soap for 20 s is the most effective method investigated when hands are either dirty or greasy. Even if not proper washing, rinsing under running water for 5 s is a cleaning procedure that may significantly reduce the probability of cross-contamination, as it removes 90% of the hands’ dirt. Although less effective than water and soap, the usage of antibacterial wipes was significantly more effective than wet wipes, indicating that they are a better choice when water and soap are not available. The results of this study enable us to inform consumers about the effectiveness of hand-cleaning procedures applied in their homes when cooking. Moreover, it can make consumers understand why, during the COVID-19 pandemic, authorities recommended washing hands as a preventive measure of infection and using an anti-bacterial hand gel or wiping hands with an antimicrobial wipe if water and soap are not available.

## 1. Introduction

The World Health Organisation [1] estimates that 600 million people—nearly 1 in 10 people in the world—get sick after eating contaminated food, and 420,000 die each year. In 2019, The European Food Safety Authority (EFSA) reported 5175 foodborne outbreaks that affected 53,383 people; inadequate consumer hygiene practices in the household being the most commonly reported source (41.3%) [2]. Foodborne diseases are preventable, and proper hand hygiene has been acknowledged as one of the most simple and adequate measures to prevent or reduce the infection rate with foodborne pathogens and the number of cases of foodborne illnesses, which usually occur through inefficient hand hygiene, cross-contamination, and transmission of certain bacteria and viruses [3,4,5,6,7,8,9,10].

Hands can act as vehicles for foodborne pathogens, and by cleaning and/or disinfecting them after touching contaminated food or surfaces, we can prevent cross-contamination and accidental ingestion [11,12,13,14].

Studies conducted on consumers during cooking have shown a low rate of compliance to hand hygiene practices, and even when performed, proper hand washing was observed in a small percentage [15,16,17,18]. According to the authorities responsible for public health, proper hand washing takes place in steps and is performed with warm water and soap for 20 s [10,19,20,21].

Washing hands with water and soap reduced the risk of foodborne illnesses during multiple contamination scenarios (i.e., transfer of *E. coli* from cutting board surface to hands, kitchen sponge to hands, etc.) by approximately 5 times compared to no handwashing [3] and reduced *Enterococcus* concentrations from farmworkers’ hands (1.6 log_10_ reduction) [22].

However, the efficacy of hand washing is influenced by several variables, such as water temperature, the use of soap, and procedure duration. As stated by the U.S. Food and Drug Administration (FDA), when washing hands, the temperature of the water should be 40 ± 2 °C. Temperature can affect the solubility or emulsification properties of some oils, and a right flow of warm water will cause the soap to lather, which could help in removing soil more efficiently from the hands [21]. Courtenay et al. [23] reported that rinsing hands with tempered water provided significantly better results in removing soil than rinsing with cold water (*p* < 0.05), while Michaels et al. [24] reported that water temperature (4.4 to 48.9 °C) did not display significant differences regarding the efficacy of hand washing. Jensen et al. [25] also indicated that water temperature (15–38 °C) had no impact on the efficacy of hand washing. Research showed that when the washing time was increased from 15 s to 30 s, the removal of *Escherichia coli* [25] and *Shigella flexneri* [26] was improved regardless of the use of antimicrobial or bland soap. A 20-s washing time with bland soap was effective in removing *Klebsiella aerogenes* (previously known as *Enterobacter aerogenes*) from hands [27].

Washing hands with water and soap has been proven to be more effective than alcohol-based solutions in reducing viruses, such as H1N1, on hands [28]. However, there are also some studies reporting that alcohol-based solutions had a higher efficacy in reducing coagulase-negative staphylococci (CoNS), *Staphylococcus aureus,* and gram-negative bacilli (GNB) [29,30] than the standard hand washing with water and soap. Washing hands in households is mentioned as one of the targeted hygiene measures that have to be taken every time the probability and level of contamination is high. The World Health Organisation (WHO), Centers for Disease Control and Prevention (CDC), and other agencies involved in protecting consumers’ health agree that, when cooking, washing hands has to be performed: before starting the cooking process and when ending it (i); after handling raw meat, poultry, seafood, and eggs (ii); before eating (iii); after touching garbage or emptying the bin (iv); after wiping counters or cleaning other surfaces with chemicals (v); after touching pets, pet food, or pet faeces (vi); after coughing, sneezing, or blowing nose (vii); after touching face, mouth, nose, or hair (vii); before and after feeding a baby or young children (viii); after using the toilet (ix); after smoking (x); before and after taking care of infected family members (xi); and after touching surfaces often touched by others (xii) [19,31,32]. Besides encouraging the following of these rules, the Royal Society of Personal Hygiene and the Institute of Food Hygiene emphasise the importance of understanding the rational for washing hands [19].

The decision to investigate the efficacy of cleaning procedures used in home kitchens came as a result of a series of visits to 75 households (15 per country) in five European countries (France, Norway, Portugal, Romania, and the UK) supported by the SafeConsume project (safeconsume.eu) to observe consumers’ behaviour in kitchens whilst handling chicken, both raw and cooked, and preparing a lettuce salad. These visits revealed that most participants applied different procedures to clean their hands (e.g., 50% rinsed hands for 5 s) [33], which raised the question: what cleaning procedure is the most effective to remove dirt (microorganisms and organic matter) from consumers’ hands?

Thus, the aim of this study was to test several cleaning procedures on the reduction of bacteria and organic dirt from hands. The results of this study will enable us to inform consumers about the effectiveness of the hand-cleaning procedure that they apply in their homes when cooking. Meanwhile, it can make consumers understand why, during the COVID-19 pandemic, authorities recommended certain hand-washing practices. 

## 2. Materials and Methods

The hand-cleaning procedures tested in this study were based on recommended hand hygiene practices as well as on observed practices from a field study of 75 European households [33]. A number of different practices were observed after touching potentially contaminated foods or areas, such as raw chicken or garbage, including: wiping hands with a dry towel or paper, rinsing in running cold or tempered water, and washing with soap or washing-up liquid in cold or tempered water. The time for washing hands varied considerably from a few seconds to more than 30 s.

On the basis of the hand-cleaning routines revealed by the SafeConsume visits, we conducted an experiment that focused on running two simulated hand-contamination scenarios and tested five hand-cleaning procedures to determine their effectiveness regarding dirt removal. Instead of measuring how clean hands were after applying a cleaning procedure, as many studies do, we evaluated the efficacy of the cleaning procedures based on the remaining dirt on the participants’ hands collected via a supplementary rinsing in a contained water volume and measured using a bioluminescence test. Quantification of dirt removal by different hand-cleaning procedures is of help in giving advice for applying adequate hygiene practices not only in kitchens but in other everyday life situations, including those when water is not available. 

### 2.1. Experimental Part

The experiment was carried out in a teaching kitchen at the Faculty of Food Science and Engineering from the Dunarea de Jos University of Galati, Romania. A convenience sample of 100 people, representing either students or technical staff, voluntarily participated. The participants were all women since they are the main food preparers in the households. Among the participants, 32% were aged 20–30 years old, 36% were 31–40 years old, 23% were 41–50 years old, and 9% were 51–60 years old. The participants were provided with the same hand-cleaning items (same type of soap, wet wipes, antibacterial wipes), washed their hands at the same sink, and dried their hands after hand washing with the same type of paper towel. Informed consent was obtained from each participant before starting the experiments and after explaining the protocol of the study.

#### 2.1.1. Procedure of Simulating Hand Contamination

To simulate bacteria and organic dirt that are specifically accumulated on hands during cooking, we used borsch (S.C. ABC S.R.L., Tecuci, Romania), a lactic fermented liquid product containing a non-pathogenic bacterium (*Lactobacillus delbrueckii* ssp. *delbrueckii*) and fine particles of wheat grains. The concentration of *L. delbrueckii* ssp. *delbrueckii* in borsch was 6 × 10^4^ mL^−1^.

To simulate greasiness, we used sunflower oil (Carrefour, Galați, Romania). 

For the first scenario of hand contamination, participants dipped each of their hands into a glass jar containing 300 mL of borsch for 20 s and then dried their hands using a forced air dryer. To simulate greasy hands in the second scenario of hand contamination, participants put 0.5 mL of sunflower oil on one of their palms and then spread the oil to both of their hands. While all 100 people participated in the ordinary dirty hands experiment, only 80 people were available for the greasy hands experiment. As there was a time gap between our experiments, 20 participants from the group involved in the first scenario were not available for the second scenario, and we did not replace them.

#### 2.1.2. Hand-Cleaning Procedures

Five hand-cleaning procedures (HCPs) were tested (see Table 1). 

For HCP3, time for rinsing was set at 5 s because the observational studies performed in SafeConsume [33,34] showed that this is often the case in real-life situations. HCP4 and HCP5 were tested as alternatives for situations when water is not available. The wet wipes (delicate wet wipes for children exclusively made for Lidl by Grande Gloria, Galati, Romania) had the following ingredients: aqua, glycerin, polysorbate 20, *Aloe barbadensis* leaf juice, *Chamomilla recutita* flower water, disodium cocoamphodiacetate, phenoxythanol, benzoic acid, sodium benzoate, dehydroacetic acid, potassium sorbate, tocopheryl acetate, sodium chloride, and fragrance. The antibacterial wipes (Hygienium, Grande Gloria, Galati, Romania) had the following active ingredients: chlorhexidine digluconate 0.4%, benzalkonium chloride 1%.

After washing, in the case of HCP 1–3, the hands were dried by patting with a paper towel.

#### 2.1.3. Quantification of Remaining Dirt on Hands after Applying Different Cleaning Procedures by the Clenching Method

In order to assess the effectiveness of HCPs on dirt removal, participants were asked to wash their hands using the reference procedure (HCP1) and then to contaminate them artificially using one of the above-mentioned procedures. For measurements, the clenching method was performed. This method is an adaptation of the hand-rinse method [35] where, instead of a plastic bag, a glass jar containing 300 mL of sterile water was used. The participants were asked to dip their right hand into the jar and rather than massaging their hands, they were asked to clench and open their fist for 20 s. As such, this method was applied: (i) after hand contamination and (ii) after hand contamination and application of a hand-cleaning procedure. The dirt released into the water was considered as total dirt when a hand-cleaning procedure was not applied and as remaining dirt when a hand-cleaning procedure was applied. The dirt that was released into the sterile water was measured using a bioluminescence test performed with Hy-Lite Liquid Test pens (Merck, Darmstad, Germany). These are ready-prepared cuvettes to measure total ATP (adenosine triphosphate) in liquid samples plus a stick for sample taking. The ATP is detected by reaction with a luciferin/luciferase reagent in a buffered solution. The HyLite Liquid Test pens, which usually are used to test the cleanliness/hygiene of liquids (e.g., CIP rinse water) in the food and beverage industry or to test biomass in water treatment applications, were used to collect the water samples immediately after the participants removed their hands from the water to avoid dirt sedimentation. The test continued according to the manufacturer’s instructions by mixing the content of the cuvette containing the reagents (luciferin/luciferase) with the water sample contained in the opposite compartment of the pen for 5 s to initiate the bioluminescence reaction. A HyLite 2 luminometer (Merck) was used for the readings. The luminometer reads the amount of light produced by each sample, and the produced light is proportional to the amount of ATP in the sample. The light detected inside the luminometer is displayed in Relative Light Units (RLU). Each participant performed each cleaning procedure once, as we were searching for data dispersion and not for repeatability. This approach happened to be beneficial to participants, as it limited the appearance of hand dryness after performing so many cleaning procedures. To limit this inconvenience, we performed the experiments on different dates, the greasy hands experiment (DH2) being organised one month later than the experiment in which hands were contaminated with borsch (DH1). 

To prove that the quantity of dirt was proportional to the RLU values, we obtained a standard curve based on different dirt loads (obtained by using different hand immersion times in borsch) and considering the intercept point as the mean HCP1—value of all participants (RLU = 45). The curve had an adequate goodness of fit, as indicated by R^2^ = 0.92 (Figure S1).

### 2.2. Statistical Analysis

The data obtained from the hand-cleaning experiment were processed with the Office Excel 19 program (Microsoft, Mountain View, CA, USA). The quartiles, interquartile range (IQR), and median were calculated for each data set. We chose to use quartiles as a measure of data spread as we expected to deal with skewed and/or data with outliers, considering the inherent variability of bioluminescence measurements and variability from person-to-person. The IQR was calculated by the difference between the third quartile (Q3) and the first quartile (Q1) to tell us about the range of the middle half of the data in the distribution and, based on this, to have a better relevance of the reported results. In order to check for outliers, we used the recommended formula [36]:

- IQR · 1.5 + Q3 > Q3 = suspected outlier
- IQR · 1.5 − Q1 < Q1 = suspected outlier

The hypothesis of normality of the data distribution was tested using the Shapiro–Wilk test. All the tests indicated that the null hypothesis is accepted, and data were normally distributed (*p* > 0.05). Moreover, the homoscedasticity test indicated equal variances of the populations. Hence, ANOVA and post-hoc Tukey method, which compares the means of the data sets (level of confidence of 95%), were applied to establish if there are significant differences between the quantities of remnant dirt associated to different hand-cleaning procedures.

### 2.3. Efficacy of Hand Cleaning Procedures

For each cleaning procedure, the efficacy was calculated using the formula:

HCP Efficacy, % = 100 − (mean value of HCP × 100/mean value of DH)

### 2.4. Graphical Representations

**Figure 1:**
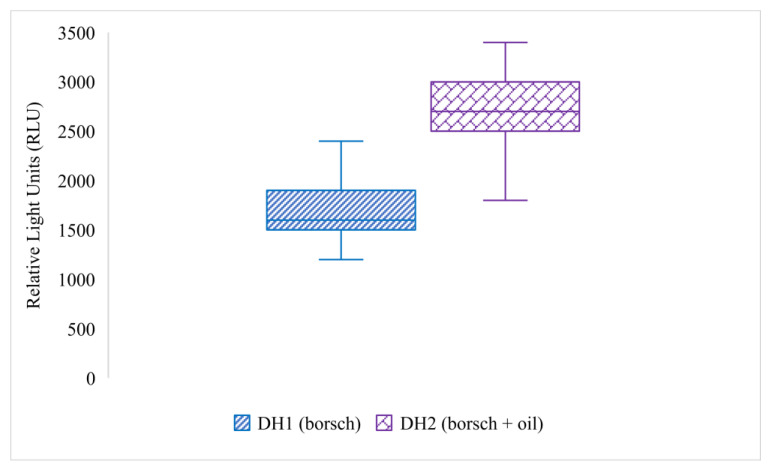
Boxplots representing the quantification of hand dirt: DH1 (**light blue**)—dirt after application of the first contamination scenario (using borsch) and DH2 (**purple**)—dirt after application of the second contamination scenario (borsch + oil).

**Figure 2:**
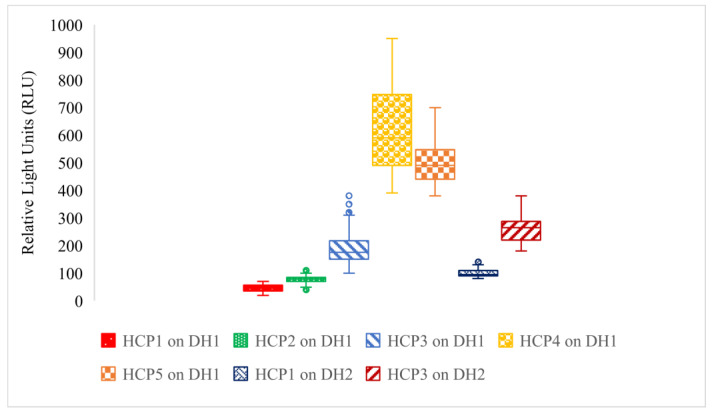
Boxplots representing the quantification of remaining dirt for HCPs on DH1 and DH2; HCP1 on DH1 (**red**)—washing hands for 20 s with running warm water (40 ± 2 °C) and soap, then drying with paper towel; HCP2 on DH1 (**green**)—washing hands for 20 s with running cold water and soap, then drying with paper towel; HCP3 on DH1 (**light blue**)—rinsing hands for 5 s with running cold water, then drying with paper towel; HCP4 on DH1 (**yellow**)—wiping hands for 20 s with wet wipes; HCP5 on DH1(**orange**)—wiping hands for 20 s with antibacterial wet wipes; HCP1 on DH2 (**dark blue**); HCP3 on DH2 (**dark red**).

The Office Excel 19 program (Microsoft) was used to obtain box plots (Figure 1 and Figure 2) representing the dispersion of dirt or remaining dirt expressed as RLU.

## 3. Results and Discussions

### 3.1. Hand Hygiene Experiment

Technical literature mentions ATP monitoring as an objective and reliable method to measure cleanliness of inanimate surfaces [37] and hands [38] if swabbing surfaces or hands takes place immediately after applying a cleaning procedure. As we wanted to measure the efficacy of different hand-cleaning procedures, we had a different approach: to quantify the dirt that stayed on hands after applying different cleaning procedures (remaining dirt) and to measure after removing from hands into a determined quantity of sterile water (method described in the previous section). Nonetheless, the greater the level of ATP, the higher the RLU value and the amount of dirt. A higher quantity of dirt indicated a lower efficacy of the hand-cleaning procedure. It is known that bioluminescence tests are able to sense, besides dirt represented by microorganisms and organic matter, the epithelial cells released when applying a cleaning procedure. However, we assumed that most of the epithelial cells were removed during cleaning hands before the planned contamination experiment, so their contribution to the RLU value obtained for total or remaining dirt is negligible.

The quantification of dirt after applying both simulated hand contamination scenarios is presented in Figure 1.

The dirt released from participants’ hands after contamination with borsch (DH1) varied from 1200 to 2400 RLU. The median associated with this data set is 1600 RLU, and the IQR is 400 RLU. Meanwhile, the dirt released from participants’ hands after contamination with borsch + oil (DH2) varied from 1800 to 3400 RLU. This data set has a median of 2700 RLU and an IQR of 500 RLU. Although the RLU values situated above the Q3 belonging to DH1 overlap the RLU values situated below the Q1 of DH2, 3/4 of the values obtained for the hands contaminated with borsch and sunflower oil are higher than those obtained for hands contaminated only with borsch, which means that oily/greasy hands necessitate a supplementary effort for cleaning them. Seven data sets are compared in Figure 2, which displays the data dispersion representing the remaining dirt on participants’ hands after applying each HCP on DH1. 

The first data set is associated with HCP1 applied on DH1. The values quantifying the dirt remaining on hands after washing with warm water and soap range from 19 to 70 RLU, with a median of 44.5 RLU and an IQR of 20.7 RLU. No outliers were present in this data set. In technical literature, values under or equaling 100 RLU obtained directly on hands signify clean hands [39,40,41].

The second data set refers to HCP2 applied on DH1. The values quantifying the remaining dirt after washing hands with cold water and soap were close to those obtained in HCP1. The values situated in the first quartile overlap those situated in the fourth quartile of the first data set, while the other goes higher but remains under 100 RLU, the limit for clean hands. The data range from 49 to 100 RLU and have a median of 78.5 RLU. The main body of data ranges from 69.5 to 84 RLU (IQR = 14.5 RLU). There are four outliers (i.e., one RLU value of 40 and three RLU values of 110) associated with this data set. 

The third data set is related to HCP3 applied on DH1. The remining dirt after rinsing hands with water for 5 s presents higher RLU values than when applying the HCP1 and HCP2 procedures, indicating a lower performance of this cleaning procedure when compared with the previous two. The RLU values range from 100 to 310 RLU, having a 4.12-times higher spread compared to HCP1 and HCP2, while the main body of data had values between 150–217.5 RLU (IQR of 67.5 RLU). The median is situated at 175 RLU. There are five outliers associated with this data set (i.e., RLU values of 320, 340, 340, 350, 380).

The fourth data set is associated with HCP4 applied on DH2. The values for the remaining dirt after wiping hands with wet tissues present a spread of the RLU values ranging from 390 to 950, an almost 2-times higher spread than HCP3, with the main data body having values between 490–747.5 RLU (IQR of 257.5 RLU). This data set has a median of 590 RLU and no outliers. 

The fifth data set relates to HCP5 applied on DH1. The values for the remaining dirt after wiping hands with antibacterial tissues range from 380 to 700 RLU, a 1.5-times higher spread than HCP3, with the main data body ranging between 440–547.5 RLU (IQR = 107.5 RLU). This data set has a median of 490 RLU and no outliers. The values from the fifth data set overlap the data from the first, second, and third quartile of the fourth data set, indicating that the performance of the two cleaning procedures is close, although HCP5 performs better. Meanwhile, both HCP4 and HCP5 had lower performances than HCP1, HCP2, and HCP3. The sixth data set refers to HCP1 applied on DH2. The values for the remaining dirt after washing hands with water and soap when having greasy hands presented a spread of values between 80–130 RLU, with the main data body of values between 90–109.5 RLU (IQR = 19.5 RLU). This data set has a median of 95 RLU and two outliers (i.e., two RLU values of 140). Although higher than the values obtained for DH1 when HCP1 is performed, more than half of the values in this data set are lower than 100 RLU, indicating that washing hands with warm water with soap for 20 s managed to release almost the entire quantity of dirt from hands even when hands are greasy. 

The seventh data set is associated with HCP3 applied on DH2. The values obtained for the remaining dirt when rinsing greasy hands with running cold water for 5 s has a spread of values between 180–380 RLU, with a main body of data with values between 220–287.5 RLU (IQR = 67.5 RLU). This data set has a median of 265 RLU and no outliers. The values obtained confirmed the fact that this cleaning method is less effective than HCP1 and less successful when grease is present.

To better evaluate the difference between the cleaning procedure performances, their efficacy was calculated. Table 2 presents the mean values and efficacy of the HCPs on different type of dirt and the significant difference if present.

Washing hands with warm water and soap for 20 s (HCP1) was the most effective hand-cleaning method. This is sustained by the fact that it had an efficacy of 97.3% on DH1 and 96.3% on DH2, being significantly better than the rest of the HCPs (*p* < 0.01; Table 2). This method functions on both types of dirt. To improve its performance when hands are greasy, its duration can be prolonged.

Washing hands with cold water and soap (HCP2) was second in place, with an efficacy of 95.4%. Nonetheless, statistically significant differences were still present between HCP1 and HCP2 (*p* < 0.01; Table 2). It is important to notice that both HCP1 applied on DH1 and DH2 had the lowest variance compared to all the other methods, while HCP2 had a similar variance as HCP1 for the DH1 procedure, but in the case of greasy hands, for DH2 procedure, it displayed a higher variance than for DH1. 

Rinsing hands with running cold water for 5 s (HCP3) was the third most effective method and had an efficacy of 88.9% on DH1 and 90.5% on DH2. This procedure was significantly less effective than HCP1 and HCP2 but proved to be more adequate at hand cleaning than wiping hands with wet/antibacterial tissues (*p* < 0.01; Table 2). The efficacy may be attributed to the power of running water in releasing the bacteria and organic dirt from hands. The highest RLU values and the highest variances were observed after participants wiped their hands with wet and antibacterial tissues (HCP4 and HCP5). However, antibacterial tissues had an efficacy of 70.8% and proved to be a better solution than the wet tissues that removed 63.8% of dirt (*p* < 0.01; Table 2). 

Hand washing with warm water and soap for 20 s proved to be the most effective procedure in both cases of contamination, displaying significant differences when compared with the other tested hand cleaning procedures. In our study, we found a small but significant difference between washing hands with warm water and soap and washing hands with cold water and soap (97.3% vs. 95.4%; *p* < 0.01). Similar results were reported by Courtenay et al. [23], where a warm rinse proved to be more effective than a cold rinse (99% vs. 94%; *p* < 0.05). In their study, the authors examined hands that were inoculated with a ground beef matrix, and the saturated fats may have been easier to remove at higher water temperatures. However, other studies reported that there were no significant differences in the efficacy of hand washing when it was performed at temperatures from 4.4 to 48.9 °C [24] or from 15 to 38 °C [25]. Authorities assert that “Cold water and warm water are equally effective at killing germs and viruses—as long as you use soap!” [42]. If not an essential factor in removing bacteria and organic dirt from hands, warm water is a comforting factor that encourages people to wash their hands more frequently than in the presence of cold water.

When we tested the efficacy of warm water and soap versus a simple rinse after contaminating hands with borsch and sunflower oil, the first method proved to be significantly more effective than the second (*p* < 0.01). Similarly, Friedrich, Julian, Kappler, Nhiwatiwa, and Moslerc [43] indicated that when washing hands, the addition of soap greatly reduced the *E. coli* counts on participants’ hands when compared with rinsing alone. Comparable results were also reported by other studies where hand washing with water and soap (average 14 s) was more effective than rinsing alone (average 12 s) in the removal of bacteria of potential faecal origin [44] and the reduction of *K. aerogenes* from the participants’ hands when comparing hand washing with soap for 20 s and rinsing alone for 5 s (*p* = 0.003) [27].

Rinsing hands for 5 s under running cold water was the third most effective procedure in the first scenario of contamination (efficacy 88.9%), being significantly better than wiping with wet/antibacterial tissues (*p* < 0.01). Similarly, rinsing hands for 5 s was also found to have an overall higher efficacy (99%) in removing pathogens, such as *Vibrio parahaemolyticus,* from surfaces like hands, knife, and cutting board, compared with wiping with a clean dish cloth (94.5%) in the study of Malcolm et al. [45].

Also, Jensen et al. [27] demonstrated that there were no significant differences (*p* > 0.05) between hand washing with soap for 20 s and rinsing alone for 20 s when beef debris was present on the participants’ hands. 

All of these suggest that an important role in the hand-cleaning process might be played by the friction of hands during rinsing, as observed by Miller, Patrick, & Ormrod, [46] and as indicated by FDA [21].

Wiping hands with wet/antibacterial wipes were the least effective cleaning procedures tested in this study, as they reduced the bacteria and organic dirt with 63.8% and 70.8%. In the study of [47], wiping hands for 60 s with a normal hand wipe was significantly less effective than hand washing with soap for 60 s. Nonetheless, in the same study, wiping hands for 60 s with an antibacterial wipe proved to be as effective as hand washing with water and soap, representing an alternative to the recommended hand-washing procedure. The relative low efficacy of wet/antibacterial wipes might be due to the fact that after transferring dirt to tissues, bacteria and organic dirt stay on the tissue and are not removed from the system until the tissue is thrown away. Although not tested in this study, using two tissues during a hand-cleaning procedure may increase the efficacy of wet/antibacterial tissues. Antibacterial tissues may be more successful than wet tissues, as they act not only as microorganism removers but as microorganism killers, too. 

When washing hands with water and soap, people tend to scrub their hands more thoroughly than when just rinsing with water. Because the steps of the recommended washing method (washing, scrubbing, rinsing, drying) take time (15–20 s), they are frequently not performed properly [48]. A quick rinse followed by shaking the hands to remove water could loosen up the microorganisms that were trapped in the ridges of the hand’s skin [48]. People, either in a hurry or because of their daily routine, tend to perform a quick rinse without soap with either cold or warm water, which is why we used it as one of the hand-cleaning procedures; this was also observed during the SafeConsume visits [33]. During cooking, few consumers were seen to perform proper hand washings, more often applying a simple hand rinse instead [15,16]. In a study that reported hand cleaning practices of consumers, actions such as rinsing or washing during food preparation were associated either with habits or the feeling of dirtiness/greasiness rather than with cross-contamination risks [33]. Among 120 participants cooking a chicken and salad dish in their kitchen, only 10% washed their hands for the recommended duration of 20 s [17]. Another study conducted by the United States Department of Agriculture within the Food Safety and Inspection Service [18] concluded that out of 383 participants, 23.9% did not use soap, and the majority (75.8%) did not rub their hands with soap for 20 s. Other researchers considered that a 10-s hand wash was more representative of what people are actually practicing [49] and that neither scrubbing hands for at least 20 s or total hand-wash time were associated with the hand’s cleanliness [43]. During food preparation, if hands are not greasy or heavily soiled, a quick rinse could prove to be effective enough. Nonetheless, since our hands often get greasy during cooking, we recommend the proper hand-washing procedure with warm water and soap. Because of its surfactant properties, soap lifts soil, grease, and microbes from the skin and when using soap, people tend to scrub their hands more vigorously than when using water alone [44,50,51]. Altogether, these findings confirm and suggest the importance of using soap when washing hands.

Antibacterial wipes were significantly more effective than wet wipes and are also adequate alternatives if soap and water are not available (i.e., when travelling) [52]. 

To our knowledge, this is the first paper that presents a hand hygiene experiment inspired by real consumer kitchen practices. These experiments indicate the efficacy of several hand-cleaning procedures by analysing the remaining dirt on the participants’ hands.

### 3.2. Study Limitations

Using borsch to simulate bacterial contamination particles is a limitation of this study, as in real situations, pathogenic bacteria may be present on hands. Using sunflower oil for the simulation of greasy hands during cooking may be another limitation as, during cooking, hands may become greasy not only because of oil but from lard, butter, or other greases. Moreover, inherent person-to-person variability happening in real-life situations contributed to the total variance of remaining dirt values.

Having just women as participants in this study may limit the data variance, as most women’s hands are smaller than men’s hands. Although the number of women involved in cooking processes prevails on the number of men, the percentage of men participate in cooking is over 20% in many countries, such as Germany, France, Spain, Denmark, Sweden [53], and the USA [54] and is on an ascending trend [54]. At the EU level, 34% is the average percent of men who are involved in cooking [53]. 

## 4. Conclusions

Washing hands with warm water and soap for 20 s is the most effective of tested methods when hands are either dirty or greasy, as it often happens during cooking poultry or meat in general. Water temperature was a significant parameter in dirt removal, as washing hands with cold water and soap was less effective than washing with warm water and soap (*p* < 0.01) but was, nonetheless, the second most effective hand cleaning procedure. Rinsing hands under running water for 5 s, a routine during meal preparation, significantly reduces the probability of cross-contamination, as it removes 90% of the hand’s dirt. However, it may not be effective enough after touching something highly contaminated with a pathogen displaying a low infectious dose (e.g., *Campylobacter* and norovirus). Antibacterial wipes were significantly more effective than wet wipes (*p* < 0.01), indicating that they are a better choice when water and soap are not available. 

The hand hygiene experiment highlights the need to inform consumers on the difference between hand-cleaning procedures. Visualisation of dirt removed from hands after applying different cleaning procedures may help consumers better understand the importance of hygiene both in kitchens or other life situations and motivate them to adopt the appropriate procedure in correlation with the type of dirt. The methodology used may inspire development of educational campaigns based on the realization of experimental activities. 

The experiments described in this manuscript may also serve as scientifically validated references for recommendations on hand washing.

## Figures and Tables

**Table 1:** Hand-cleaning procedures.

Hand Cleaning Procedures (HCPs)	Description of HCPs
HCP 1	Washing hands for 20 s with running warm water (40 ± 2 °C) and bland soap, then drying with paper towel (this procedure was considered as reference)
HCP 2	Washing hands for 20 s with running cold water and bland soap, then drying with paper towel (cold water replaced warm water from the reference)
HCP 3	Rinsing hands for 5 s with running cold water, then drying with paper towel
HCP 4	Wiping hands for 20 s with wet wipe
HCP 5	Wiping hands for 20 s with antibacterial wet wipe

**Table 2:** Efficacy of the tested hand-cleaning procedures on DH1 and DH2 scenarios.

RLU on Hands	Mean Value (SEM), RLU	Efficacy, %	Grouping Letter *
**DH1 contamination**	1708 (25.6)		
Warm water + soap	45.1 (1.3)	97.3	A
Cold water + soap	77.2 (1.2)	95.4	B
Rinse	188 (6)	88.9	C
Wipe with wet tissue	618 (14.3)	63.8	D
Wipe with antibacterial tissue	498.6 (8.4)	70.8	E
**DH2 contamination**	2725 (35.6)		
Warm water + soap	100.1 (1.6)	96.3	F
Rinse	258.5 (4.8)	90.5	G

DH1, first scenario of contamination (borsch); DH2, second scenario of contamination (borsch + oil); RLU, Relative Light Units; SEM, Standard Error of the Mean; * Means that do not share the same letter are significantly different at *p* < 0.01.

## Data Availability

The datasets analysed in this study are available from the corresponding author on reasonable request.

## Supplementary Materials

The following are available online at https://www.mdpi.com/article/10.3390/ijerph18168828/s1, Figure S1: Calibration curve for simulated dirtiness.
