# Supplementary figures and images for: Efficacy of Removing Bacteria and Organic Dirt from Hands—A Study Based on Bioluminescence Measurements for Evaluation of Hand Hygiene When Cooking
Source: Int J Environ Res Public Health. 2021 Aug 21;18(16):8828. doi: 10.3390/ijerph18168828 (PMC8394668; doi:10.3390/ijerph18168828)

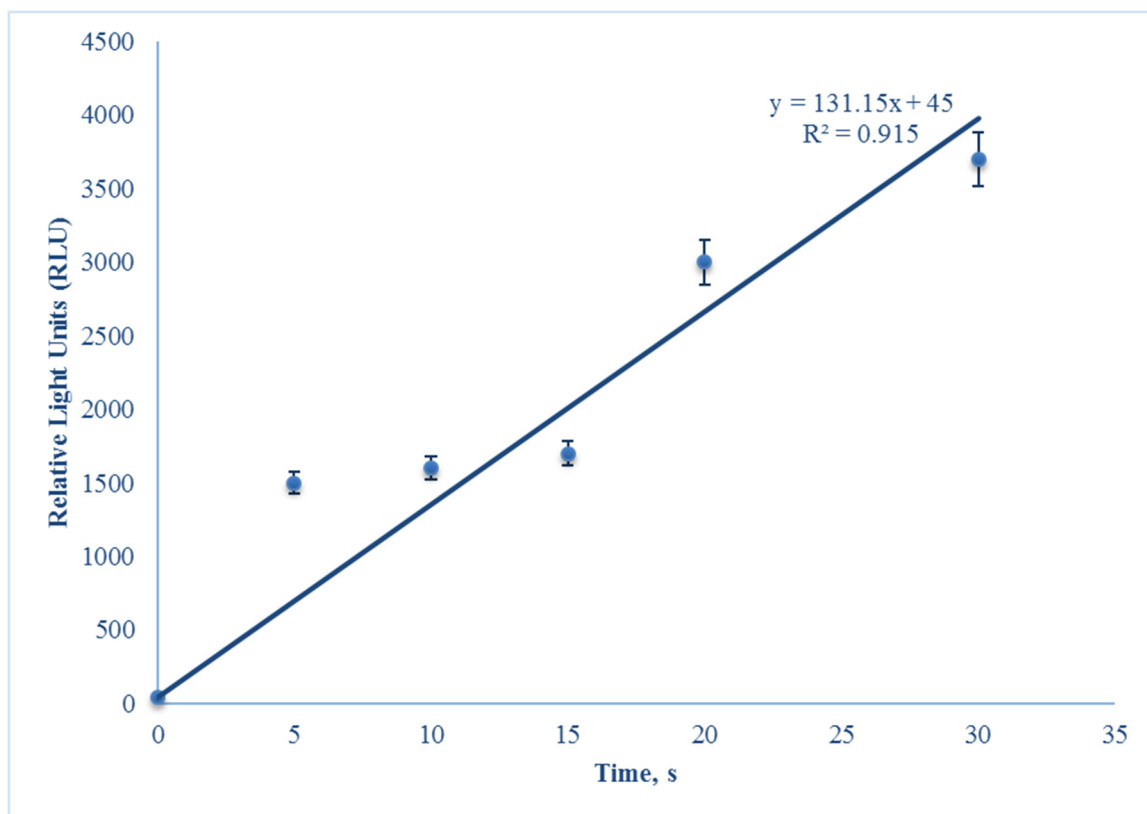

**Figure S1.** Calibration curve for simulated dirtiness.

Supplement: Supplementary file 1 [file ijerph-18-08828-s001.zip › ijerph-1326004-SI.pdf]
